# Editorial: Recent advances in cervical cancer radiotherapy

**DOI:** 10.3389/fonc.2023.1144797

**Published:** 2023-02-15

**Authors:** John Michael Varlotto, Gene A. Cardarelli

**Affiliations:** ^1^ Department of Oncology, Marshall University Chief of Radiation Oncology, Edwards Comprehensive Cancer Center, Huntington, WV, United States; ^2^ Department of Radiation Oncology, Warren Alpert Medical School, Rhode Island Hospital, Brown University, Providence, RI, United States

**Keywords:** cervical cancer, anti-angiogenic therapy, vaginal microbiome, interstitial HDR brachytherapy, lexicographic optimization

It is a great pleasure to serve as Editors for the Frontiers e-blook, Recent Advances in Cervical Cancer.

Despite the availability of good screening and HPV vaccine strategies, cervical cancer remains the fourth most common cancer in women and still causes 570,000 new cases and 311,000 deaths globally as reported in 2018 (1). Nearly 90% of cervical cancer deaths occur in developing countries, with India and China accounting for 35% of the total cervical cancer burden (2). Meanwhile in the United States, the CDC has recommended to stop screening for most women age 65 years or older. However, The United States may be underscreening elderly women as shown by a recent retrospective review from the California Cancer Registry which demonstrated that 17.4% of cervical cancers were in women ≥ 65 yrs or older (3).

In 1999, 3 simultaneous prospective, randomized trials demonstrated the efficacy of concomitant cisplatin-based chemo/radiation (4–6) for locally advanced cervical cancer. Since this time, there has only been a refinement of our approach to the radiotherapeutic treatment for cervical cancer *via* the use of IMRT(proven in the adjuvant setting (7, 8), and the MRI-planning for cervical HDR brachytherapy as per EMBRACE (9), but there has been no recent trial that has shown a further improvement in survival in locally advanced cervical cancer.

Recently, the NRG Oncology Group reported the results of the phase I/Ib NRG-GY017 (10) and showed that the addition of atezolizumab to concurrent chemo/radiation in node-positive cervical cancer was feasible. Additionally, there was increase in peripheral blood T-cell receptor (TCR) clonal expansion and expansion of tumor-associated T-cell clones between the start of treatment and day 21 of concurrent chemo/radiation. Patients with higher pre-treatment TCR diversity had increased likelihood of biopsy-proven complete pathologic response. However, the role of immunotherapy remains to be determined.

This special issue of Frontiers will allow the reader to review cutting-edge research in radiation planning/treatment delivery and to assess valuable clinical studies on the use of re-irradiation of locally recurrent cervical cancer with interstitial HDR brachytherapy, the development of prognostic tools from conventional PETCT Scans, the optimal timing of radiotherapy as per the circadian cycle, the use of an anti-angiogenic agent concurrently with chemo/radiotherapy, and the changes of the vaginal microbiome during radiotherapy.

Five studies in this special issue have shown potential improvements in the efficacy and speed of treatment planning. The dosimetric study by Trivellato et al. compared plans for locally advanced cervical cancer using lexicographic optimization vs standard IMRT optimization. The lexicographic optimization mimics the conversations concerning treatment planning between the Radiation Oncologists and the treatment planning team by using inviolable dose constraints and a hierarchical list of objectives. By utilizing this technology, the median treatment planning/optimization period was reduced from 4 hours to just over 1 hour while increasing planning tumor volume coverage and plan complexity and offering similar organ-at-risk dose constraints. Researchers from Shanghai Sixth People’s Hospital have proposed and verified a method of machine learning for optimization of cervical HDR-brachytherapy which allows for reduction of normal tissue doses and more efficient planning time (Li et al.). Investigators from The First Affiliated Hospital of Xi’an noted that an atlas-based auto-segmentation of volumes undergoing radiotherapy can be performed quickly and accurately for tumor and normal tissue volumes with the exception of rectum (Li et al.). Wu et al. from University of Science and Technology of China Anhui Provincial Hospital used a scatter-beam correction method in order to facilitate improved image quality of cone-beam ct scans to improve dosimetric accuracy of cervical brachytherapy. Zhou et al. from Affiliated Hospital of Southwest Medical University used a support vector machine model to predict the D2cm3 for the bladder, rectum, sigmoid colon, and small intestine in patients undergoing cervical cancer brachytherapy.

Two studies investigated potential prognostic factors that could, if proven in follow-up studies, help identify high-risk populations for treatment failure. Wang et al. from Peking Union Medical College Hospital (CAMS) assessed tumor/nodal metabolic parameters on PET/CT and their association with outcomes in 125 consecutive patients with locally advanced cervical cancer. This study noted that total lesion glycolysis and SUV max in the primary tumor volume and cervical lymph nodes, respectively are associated with overall survival, disease-free survival and distant metastasis-free survival. If confirmed, the results can hopefully identify patients who can benefit from treatment intensification. There have been intriguing studies of the microbiome and their effects of therapeutic outcomes of patients undergoing cancer therapy (11, 12). Jiang et al. demonstrated that the vaginal microbiome was different in 20 cervical cancer patients as compared to six healthy controls. Furthermore, the relative abundance of certain vaginal microbes increased over time. It will be interesting to see if follow-up studies can assess whether certain microbes are associated with treatment efficacy and if the microbial environment can be altered to improve outcomes in patients with cervical cancer undergoing radiotherapy.

Three clinical studies have demonstrated interesting strategies to improve outcomes for patients with cervical cancer undergoing radiotherapy. Based upon the concept that tumor and normal tissue both follow a circadian rhythm (13–15), Wang et al. report a very interesting prospective, randomized, multi-institutional clinical trial that randomized patients with locally advanced cervical cancer to morning (9:00–11:00 AM) or evening radiotherapy (7:00–9:00 PM) radiotherapy. Although the efficacy of therapy was similar in both groups, the evening group experienced less radiation enteritis and radiation diarrhea at the expense of a higher incidence of bone marrow suppression and hematologic toxicity. Ren et al. evaluated HDR interstitial brachytherapy for consecutive patients with locally recurrent cervical cancer in a previously irradiated area. Although the complete response rate was 56.5%, the 4-year post-relapse survival (PRS) rate was only 27.1% and 9 of the 23 patients(39.1%) experienced grade 3-4 late toxicity. Their approach to re-irradiation was quite daring because the average clinical tumor volume was quite large 82.9 cm3 (range: 26.9–208.3 cm3). Nevertheless, despite the high risk of serious toxicity, the authors noted that local tumor control was associated with overall survival. Authors from The First Affiliated Hospital of Anhui Medical University report an exciting prospective, randomized single-institution trial using the anti-angiogenic agent, Endostar, with concurrent chemo/radiation as compared to the same concurrent chemoradiotherapy regimen without Endostar. Endostar is a recombinant human vascular endostatin pharmaceutical agent that was made by adding 9 amino acids to the original endostatin molecule (16). Although the concurrent chemotherapy used was relatively non-standard(cisplatin, paclitaxel), the experimental arm yielded significantly higher complete response rates and a lower incidence of nausea at the expense of higher incidences of neutropenia, hypertension, and infection (Shu et al.). Although short and long-term survival are not available, the follow-up of this exciting trial is eagerly anticipated.

We hope that this issue and the involved studies promote further advances and refinement to the radiotherapeutic approach to cervical cancer. Furthermore, it is hoped that further evaluation of the sequencing of radiotherapy with anti-angiogenic agents and immunotherapy can start improving the survival of cervical cancer patients for the first time in over 23 years.

## Author contributions

JMV provided the majority of the written manuscript. GC reviewed and provided edits as needed for submission. Both served as research editors for the Cervical Cancer research edition. All authors contributed to the article and approved the submitted version.
